# Strontium Attenuates Hippocampal Damage via Suppressing Neuroinflammation in High-Fat Diet-Induced NAFLD Mice

**DOI:** 10.3390/ijms241210248

**Published:** 2023-06-16

**Authors:** Shuai Wang, Fangyuan Zeng, Yue Ma, Jiaojiao Yu, Chenyao Xiang, Xiao Feng, Songlin Wang, Jianguo Wang, Shanting Zhao, Xiaoyan Zhu

**Affiliations:** College of Veterinary Medicine, Northwest A&F University, Yangling, Xianyang 712100, China; ws13785365876@163.com (S.W.);

**Keywords:** strontium, hippocampal damage, neuroinflammation, non-alcoholic fatty liver disease, endoplasmic reticulum stress, hippocampal synaptic plasticity

## Abstract

Non-alcoholic fatty liver disease (NAFLD) leads to hippocampal damage and causes a variety of physiopathological responses, including the induction of endoplasmic reticulum stress (ERS), neuroinflammation, and alterations in synaptic plasticity. As an important trace element, strontium (Sr) has been reported to have antioxidant effects, to have anti-inflammatory effects, and to cause the inhibition of adipogenesis. The present study was undertaken to investigate the protective effects of Sr on hippocampal damage in NAFLD mice in order to elucidate the underlying mechanism of Sr in NAFLD. The mouse model of NAFLD was established by feeding mice a high-fat diet (HFD), and the mice were treated with Sr. In the NAFLD mice, we found that treatment with Sr significantly increased the density of c-Fos^+^ cells in the hippocampus and inhibited the expression of *caspase-3* by suppressing ERS. Surprisingly, the induction of neuroinflammation and the increased expression of inflammatory cytokines in the hippocampus following an HFD were attenuated by Sr treatment. Sr significantly attenuated the activation of microglia and astrocytes induced by an HFD. The expression of phospho-*p38*, *ERK*, and *NF-κB* was consistently significantly increased in the HFD group, and treatment with Sr decreased their expression. Moreover, Sr prevented HFD-induced damage to the ultra-structural synaptic architecture. This study implies that Sr has beneficial effects on repairing the damage to the hippocampus induced by an HFD, revealing that Sr could be a potential candidate for protection from neural damage caused by NAFLD.

## 1. Introduction

Non-alcoholic fatty liver disease (NAFLD) is a potentially progressive liver disease, which can lead to cirrhosis, hepatocellular carcinoma, liver transplantation, and even death [1]. Increasing evidence suggests that NAFLD not only affects liver function but also causes a variety of extrahepatic manifestations, such as depression, cognitive impairment, Alzheimer’s disease, and dementia [2]. Caused by the interactions of genetics, lifestyle, and diet, NAFLD is a multi-factorial disease [3]. A high-fat diet (HFD) is regarded as one of the most vital environmental factors that has resulted in the global NAFLD epidemic, and it also leads to type II diabetes and neurodegenerative diseases [4,5,6]. A long-term HFD, which is closely associated with NAFLD and NAFLD-related diseases, not only causes an abnormally high accumulation of body fat and metabolic disorders but also impairs the function of the central nervous system (CNS) [7].

Strontium (Sr) is a chemical element in the same family as the better-known elements calcium (Ca), barium, and radium of the alkaline earth metals. As an important trace element, Sr is beneficial for the maintenance of life. Previous research on Sr has primarily focused on its effects on bone tissue metabolism and its application in osteoporosis treatment [8,9,10]. Likewise, it has been reported to have antioxidant effects, to have anti-inflammatory effects, and to cause the inhibition of adipogenesis. For instance, the protective effects of Sr were mainly mediated by inhibiting the inflammatory responses via down-regulating the *NF-κB* pathway [11]. Strontium ranelate attenuated the elevation of *β-catenin* induced by *IL-1β*, thus reducing the inflammatory reaction [12]. Morphological damage, a decreased serum testosterone level, and apoptosis in the testes can be partially alleviated by strontium fructose-1,6-diphosphate via suppressing the increased oxidative stress [13]. Additionally, Vidal C and colleagues found that Sr may inhibit adipogenesis by activating the peroxisome proliferator-activated receptors in the adipocytes [14]. These reports indicated that Sr potentially plays an active role under various adverse factors.

It is well known that the hippocampus plays a crucial role in the functions of learning and memory. Hippocampus-dependent learning and memory appear to be particularly vulnerable to HFD-induced damage [15]. Animal studies have suggested that an HFD can result in NAFLD as well as deficits in the learning and memory processes that are dependent on the hippocampus [6,16,17]. An HFD can cause endoplasmic reticulum stress (ERS), inflammation, and lipidic toxicity, playing a major role in the development of NAFLD [7,18,19]. As homologous elements, Sr and Ca have similar chemical properties and biological characteristics. Some studies have shown that Sr can replace Ca in some physiological processes, such as in the process leading to neurohypophysis hormone secretion and in the process of transmitter release at the neuromuscular junction [20,21]. Moreover, a previous report described that Ca could alleviate neuronal dysplasia in the hippocampus [22]. Therefore, we hypothesized that Sr may help prevent and treat the hippocampal damage caused by NAFLD, although there is a lack of available studies on the effects and mechanisms of Sr in hippocampal damage.

This research was undertaken to investigate the protective effects of Sr on HFD-induced hippocampal damage in an NAFLD mouse model. Here, we reported that Sr inhibited apoptosis, ERS, and neuroinflammation and restored hippocampal synaptic plasticity. Our results demonstrated that Sr had beneficial effects on repairing the hippocampal damage induced by an HFD in an NAFLD mouse model, revealing that Sr could perform as a potential candidate to protect from neural lesions caused by NAFLD. Meanwhile, these results also open a way to provide new ideas for preventing and treating HFD-induced hippocampal damage.

## 2. Results

### 2.1. The NAFLD Mouse Model Induced by an HFD Was Successfully Established

**Figure 1:**
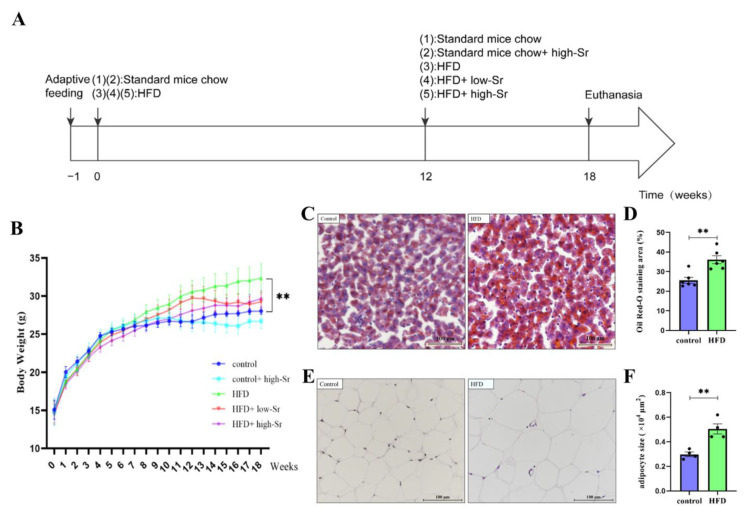
Methods and results of establishing the NAFLD model. (**A**) The NAFLD model was established according to the method shown. (**B**) The body weight of control, control + high Sr, HFD, HFD + low Sr and HFD + high Sr groups at the end of every week of treatment is shown (*n* = 8–12 per group). (**C**) Representative images of hepatic lipid deposition stained with Oil Red O in 8 μm frozen sections of control and HFD groups. (**D**) Percentage of Oil Red-O staining area quantitative analysis (*n* = 6 per group). (**E**) Representative images of epididymal adipose stained with H&E in 20 μm paraffin sections of control and HFD groups. (**F**) Quantification of the average adipocyte size of epididymal fat tissue (*n* = 4 per group). Data are presented as mean ± SEM. ** *p* < 0.01.

Firstly, the NAFLD model was established according to the method shown in Figure 1A. There was no significant difference among the groups in initial body weight, whereas the body weight of the HFD-fed mice was higher than that in all the standard-mouse-chow-fed groups after 12 weeks of HFD administration (Figure 1B). At the end of the experiment (18 weeks), the HFD group had significant weight gain in comparison with the control group (*p* = 0.0080; Figure 1B). In the Oil Red O analysis performed to assess hepatic fat accumulation, more lipid droplets with darker staining were observed in the HFD group than in the control group (Figure 1C). There was a significant difference in the percentage of Oil Red-O staining area between these two groups (*p* = 0.0021; Figure 1D), suggesting that the hepatic fat accumulation in the HFD group was aggravated. Furthermore, the epididymal adipocyte size in the HFD group was markedly enlarged compared to that in the control group (Figure 1E), and the quantitative analysis showed the consistent result (*p* = 0.0040; Figure 1F). These characteristics indicated that the NAFLD mouse model was successfully constructed.

### 2.2. Sr Increased the Expression of c-Fos in HFD-Fed Mice

**Figure 2:**
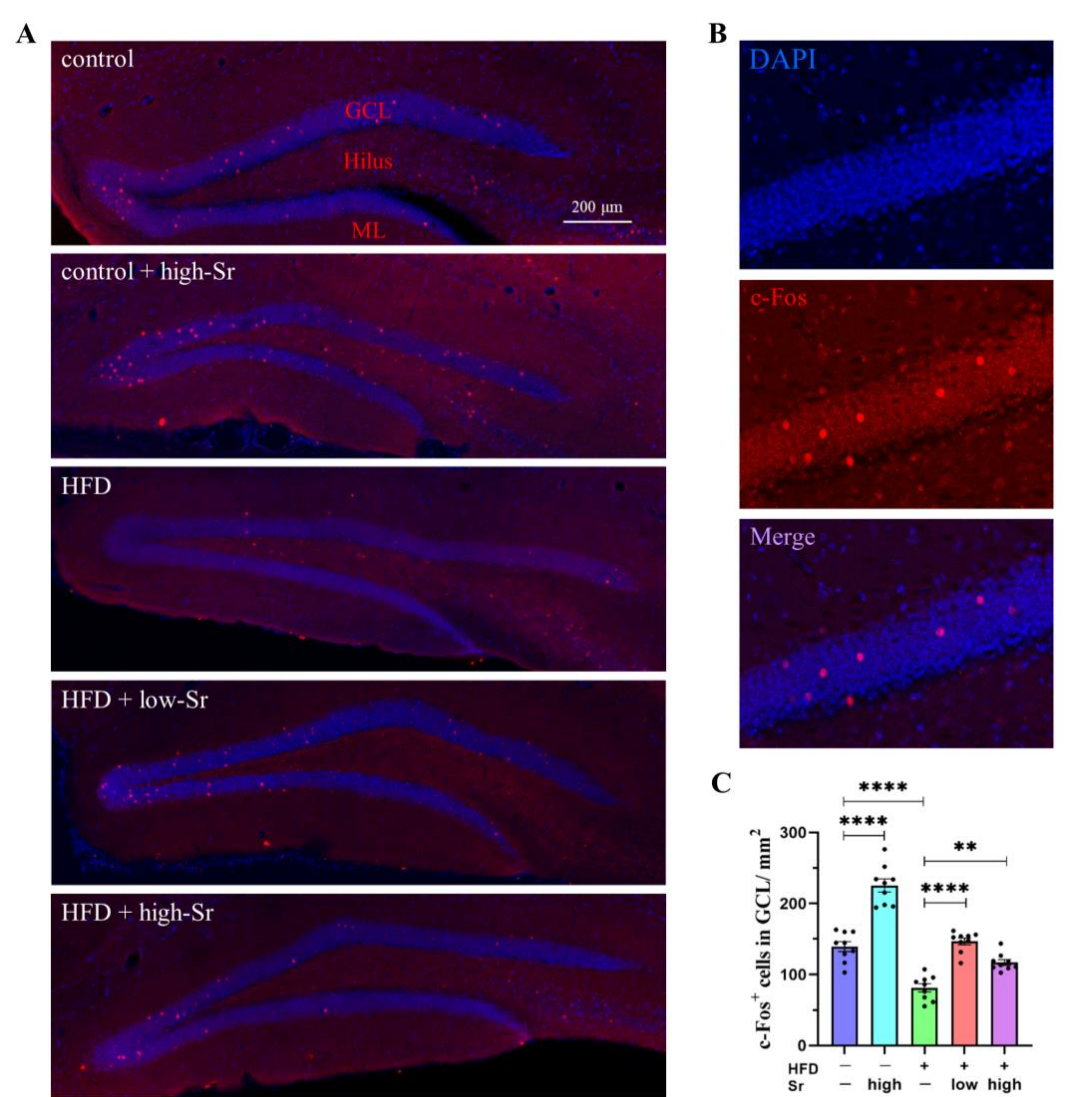
Sr enhanced the expression of c-Fos in the hippocampal GCL. (**A**) Representative images of immunostaining of c-Fos (red) in the hippocampus. The sections were counterstained with DAPI (blue) for nuclei to show the architecture of the dentate gyrus (DG). DG can be roughly seen in GCL, ML, and hilus. (**B**) Merge represents a composite of both channels. (**C**) Quantitation of c-Fos+ cells staining in the GCL of mice from each group (*n* = 9 per group). Data are presented as mean ± SEM. ** *p* < 0.01; **** *p* < 0.0001.

As an activator protein 1 transcription factor, it is well known that c-Fos is implicated in the regulation of cell proliferation, survival, and apoptosis [23]. We initially investigated whether Sr affected c-Fos expression in the hippocampi of the mice mediated by an HFD. The expression of c-Fos was evaluated through immunofluorescence staining with a specific antibody, and the c-Fos^+^ cells in the granule cell layer (GCL) of the hippocampus were counted. The results showed that an HFD considerably reduced the density of c-Fos^+^ cells in the GCL (*p* < 0.0001; Figure 2C). However, Sr inhibited the reduction in c-Fos^+^ cells in the GCL following a long-term HFD (low Sr: *p* < 0.0001) (high Sr: *p* = 0.0029) (Figure 2C). Interestingly, in the control group with high Sr, the density of c-Fos^+^ cells was greatly increased in the GCL (*p* < 0.0001; Figure 2C). These results demonstrated that Sr could increase the expression of c-Fos in the hippocampal GCL of the HFD-fed mice.

### 2.3. Sr Suppressed HFD-Induced Apoptosis by Inhibiting ERS

**Figure 3:**
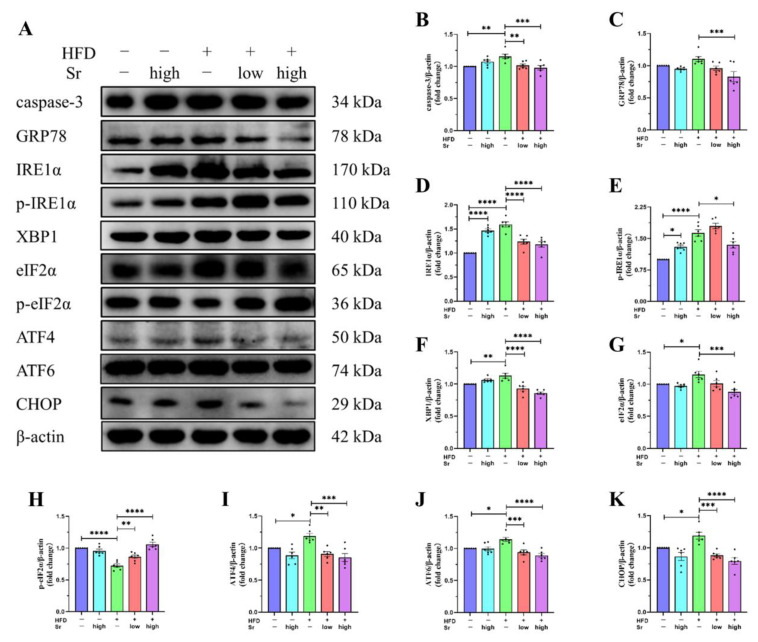
Sr restrained the HFD-induced apoptosis by altering expression levels of proteins related to the ERS pathway. (**A**) Western blot analysis of caspase-3, GRP78, IRE1α, p-IRE1α, XBP1, eIF2α, p-eIF2α, ATF4, ATF6, CHOP, and β-actin. (**B**–**K**) Relative protein expression of caspase-3 (**B**), GRP78 (**C**), IRE1α (**D**), p-IRE1α (**E**), XBP1 (**F**), eIF2α (**G**), p-eIF2α (**H**), ATF4 (**I**), ATF6 (**J**), and CHOP (**K**) in the hippocampi of each group of mice was examined through Western blotting (*n* = 6 per group). Data were normalized with respect to the band of β-actin: the expression of target protein = the intensity of target protein band/the intensity of β-actin band. Results are shown as the ratio of the experimental group to the control group, and the values of the control group were taken as 1. All data are presented as mean ± SEM. * *p* < 0.05, ** *p* < 0.01, *** *p* < 0.001, and **** *p* < 0.0001.

Having demonstrated that an HFD inhibited the expression of c-Fos associated with apoptosis, we investigated whether an HFD could induce apoptosis in the hippocampus. Since *caspase-3* is considered to be the primary effector apoptosis molecule [24], its activity was examined to confirm apoptosis. We found that the *caspase-3* protein level visibly increased in the HFD group (*p* = 0.0030; Figure 3B), and treatment with Sr decreased its expression significantly (low Sr: *p* = 0.0089) (high Sr: *p* = 0.0008) (Figure 3B). Previous studies indicated that an HFD could result in hippocampal damage through ERS-induced cell apoptosis [25,26,27]. Although the HFD group showed a trend towards a higher *GRP78* level in the hippocampus when compared to the control group, the level in the HFD group with high Sr decreased significantly compared to that in the HFD group (*p* = 0.0010; Figure 3C). To further investigate whether the ERS response is actually induced by an HFD, we analyzed the expression of several ERS-inducible unfolded protein response (*UPR*) proteins. As expected, the levels of *IRE1α*, *p-IRE1α*, *XBP1*, *eIF2α*, *ATF4*, and *ATF6* increased significantly in the HFD group compared to those in the control group (*IRE1α*: *p* < 0.0001) (p-*IRE1α*: *p* < 0.0001) (*XBP1*: *p* = 0.0099) (*eIF2α*: *p* = 0.0466) (*ATF4*: *p* = 0.0499) (*ATF6*: *p* = 0.0201) (Figure 3D–G,I,J). Sr significantly decreased the levels of the above proteins in the HFD mice (low Sr: (*IRE1α*: *p* < 0.0001) (*XBP1*: *p* < 0.0001) (*ATF4*: *p* = 0.0014) (*ATF6*: *p* = 0.0006)) (high Sr: (*IRE1α*: *p* < 0.0001) (*p-IRE1α*: *p* = 0.0220) (*XBP1*: *p* < 0.0001) (*eIF2α*: *p* = 0.0002) (*ATF4*: *p* = 0.0002) (*ATF6*: *p* < 0.0001)) (Figure 3D–G,I,J). High Sr without HFD provoked an elevation in the *IRE1*α and *p-IRE1α* levels (*IRE1α*: *p* < 0.0001) (*p-IRE1α*: *p* = 0.0123) (Figure 3D,E). Compared with the control group, the expression of *p-eIF2α* significantly decreased in the HFD group (*p-eIF2α*: *p* < 0.0001) (Figure 3H), while the level of *p-eIF2α* significantly increased (to different extents) with the treatment of low Sr or high Sr (low Sr: *p* = 0.0095) (high Sr: *p* < 0.0001) (Figure 3H). The transcription factor *CHOP* was reported to be a molecule involved in ERS-induced apoptosis that plays an important role in the induction of apoptosis [28,29]. We found that the protein level of *CHOP* increased significantly in the HFD group (*p* = 0.0471; Figure 3K), and treatment with Sr markedly decreased its expression (low Sr: *p* = 0.0005) (high Sr: *p* < 0.0001) (Figure 3K). Overall, these results showed that Sr could suppress HFD-induced apoptosis by inhibiting ERS.

### 2.4. Sr Inhibited the Production of Inflammatory Cytokines in HFD-Fed Mice

**Figure 4:**
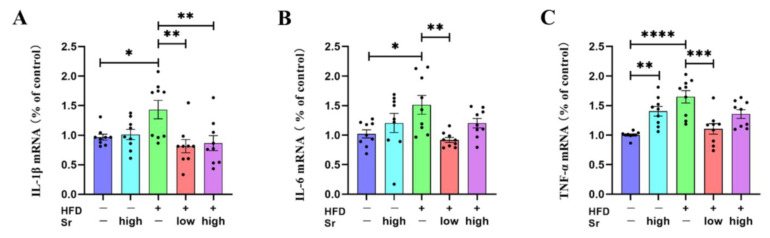
Sr inhibited the HFD-induced inflammatory gene expression in the hippocampus. (**A**–**C**) IL-1β (**A**), IL-6 (**B**), and TNF-α (**C**) mRNA expression levels in the hippocampi of each group of mice were examined through real-time RT-PCR (*n* = 9 per group). Data are presented as mean ± SEM. * *p* < 0.05, ** *p* < 0.01, *** *p* < 0.001, and **** *p* < 0.0001.

Accumulating studies have demonstrated that prolonged ERS could regulate the inflammatory responses that mediate the production of cytokines [30,31]. An HFD can not only induce neuroinflammation but also increase the expression of inflammatory cytokines [32,33]. To determine whether Sr could suppress the HFD-induced increase in inflammatory cytokines in the hippocampus, an RT-PCR analysis was performed to assess the levels of *IL-1β*, *IL-6*, *TNF-α*, and *GAPDH*. The results showed that HFD significantly increased the mRNA levels of *IL-1β*, *IL-6*, and *TNF-α* in the hippocampus (*IL-1β*: *p* = 0.0410) (*IL-6*: *p* = 0.0383) (*TNF-α*: *p* < 0.0001) (Figure 4A–C). Low Sr significantly reduced the expression levels of the above inflammatory cytokines compared with the HFD group (*IL-1β*: *p* = 0.0031) (*IL-6*: *p* = 0.0066) (*TNF-α*: *p* = 0.0002) (Figure 4A–C). Although the mRNA level of *IL-1β* in the HFD group with high Sr was decreased significantly compared to the HFD group, high Sr without an HFD increased the mRNA level of *TNF-α* compared to the control group (*IL-1β*: *p* = 0.0077) (*TNF-α*: *p* = 0.0079) (Figure 4A,C). These results suggested that Sr could be capable of inhibiting the HFD-induced production of inflammatory cytokines.

### 2.5. Sr Suppressed the Activation of Microglia and Astrocytes in the Hippocampus Induced by an HFD

**Figure 5:**
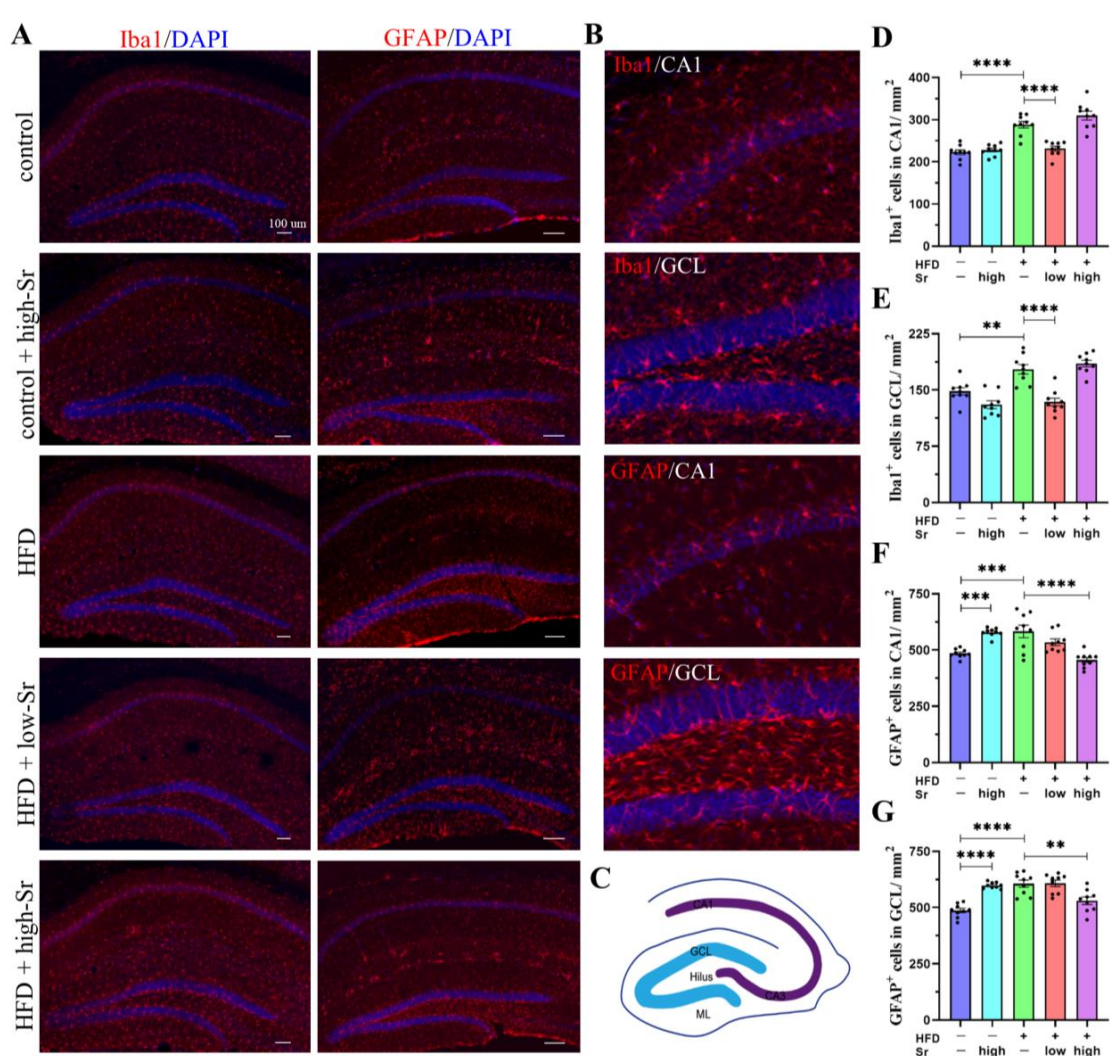
Sr inhibited the activation of microglia and astrocytes induced by HFD. (**A**) Representative images of immunostaining of Iba1 or GFAP (red) in the hippocampus. The sections were counterstained with DAPI (blue) for nuclei to show the architecture. Hippocampus can be roughly seen in GCL, ML, hilus, CA1, and CA3. (**B**) Merge represents a composite of both channels. (**C**) Schematic diagram of the hippocampal region; Blue region represents GCL, and purple region represents the central part of hippocampus. (**D**–**G**) Quantitation of Iba1^+^ cells and GFAP^+^ cells staining in the CA1 or GCL of mice from each group (*n* = 9 per group). Data are presented as mean ± SEM. ** *p* < 0.01, *** *p* < 0.001, and **** *p* < 0.0001.

The activation of microglia and astrocytes is thought to be involved in the increase in inflammatory cytokines [34,35,36]. The increase in the number of Iba1^+^ cells and GFAP^+^ cells was viewed as the marker of the activation of microglia and astrocytes [37,38]. According to our results, the number of Iba1^+^ cells and GFAP^+^ cells in the HFD group increased significantly compared with the control group (Iba1: (CA1: *p* < 0.0001) (GCL: *p* = 0.0020)) (GFAP: (CA1: *p* = 0.0005) (GCL: *p* < 0.0001)) (Figure 5D–G). Low Sr treatment significantly decreased the number of Iba1^+^ cells in the CA1 and GCL (CA1: *p* < 0.0001) (GCL: *p* < 0.0001) (Figure 5D,E). High Sr treatment not only decreased the number of GFAP^+^ cells in the HFD group (CA1: *p* < 0.0001) (GCL: *p* = 0.0016) (Figure 5F,G) but also substantially increased the number of GFAP^+^ cells in the control group (CA1: *p* = 0.0010) (GCL: *p* < 0.0001) (Figure 5F,G). These findings demonstrated that the activated microglia and astrocytes in the hippocampus induced by an HFD can be inhibited by Sr.

### 2.6. Sr Inhibited the Activation of the TLR4/p38 MAPK/ERK and NF-κB Pathways Induced by an HFD

**Figure 6:**
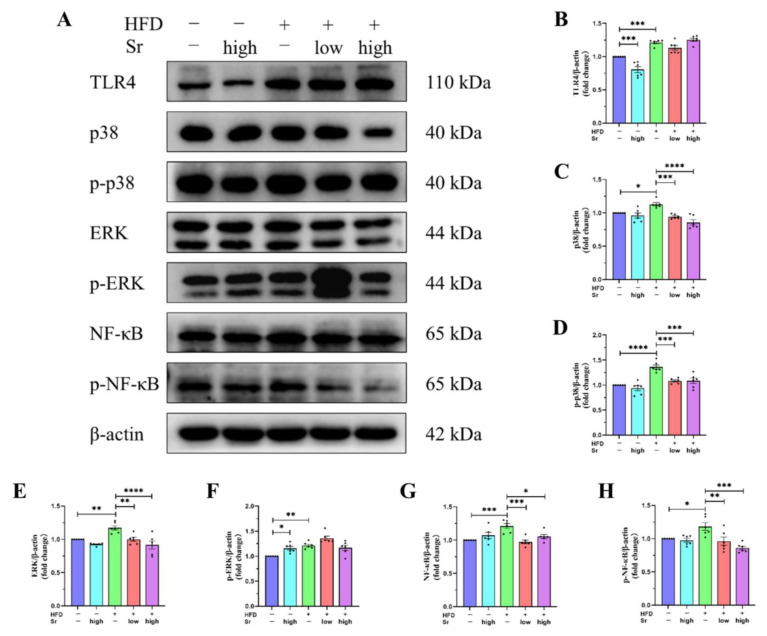
Suppressive effects of Sr on HFD-induced activation of the TLR4/p38 MAPK/ERK and NF-κB pathways. (**A**) Representative bands of TLR4, p38, p-p38, ERK, p-ERK, NF-κB, p-NF-κB, and β-actin expression detected through Western blotting. (**B**–**H**) Densitometric quantification of TLR4, p38, p-p38, ERK, p-ERK, NF-κB, and p-NF-κB proteins were performed and expressed as ratios of TLR4 (**B**), p38 (**C**), p-p38 (**D**), ERK (**E**), p-ERK (**F**), NF-κB (**G**), and p- NF-κB (**H**) to β-actin in the hippocampi of each group of mice that were examined (*n* = 6 per group). Data were normalized with respect to the band of β-actin: the expression of target protein = the intensity of target protein band/the intensity of β-actin band. Results are shown as the ratio of the experimental group to the control group, and the values of the control group were taken as 1. All data are presented as mean ± SEM. * *p* < 0.05, ** *p* < 0.01, *** *p* < 0.001, and **** *p* < 0.0001.

Although we demonstrated that Sr can attenuate the HFD-induced secretions of inflammatory cytokines and the massive activation of microglia and astrocytes, the potential mechanisms by which Sr suppresses neuroinflammation remain unclear. To investigate the possible mechanisms, the levels of *TLR4*, *p38*, *p-p38*, *ERK*, *p-ERK*, *NF-κB*, and *p-NF-κB* in the hippocampus were determined (Figure 6A). The results revealed that the levels of the above inflammation-related proteins were visibly higher in the HFD group than those in the control group (*TLR4*: *p* = 0.0002) (*p38*: *p* = 0.0205) (*p-p38*: *p* < 0.0001) (*ERK*: *p* = 0.0088) (*p-ERK*: *p* = 0.0025) (*NF-κB*: *p* = 0.0008) (*p-NF-κB*: *p* = 0.0394) (Figure 6B–H). However, Sr treatment restrained the expression of *p38*, *p-p38*, *ERK*, *NF-κB*, and *p-NF-κB* in the HFD group (low Sr: (*p38*: *p* = 0.0006) (*p-p38*: *p* = 0.0003) (*ERK*: *p* = 0.0085) (*NF-κB*: *p* = 0.0002) (*p-NF-κB*: *p* = 0.0082)) (high Sr: (p38: *p* < 0.0001) (*p-p38*: *p* = 0.0003) (*ERK*: *p* < 0.0001) (*NF-κB*: *p* = 0.0147) (*p-NF-κB*: *p* = 0.0001)) (Figure 6C–E,G,H). As seen in Figure 6B,F, in the control group, the expression of *TLR4* was diminished by high Sr treatment, but the expression of *p-ERK* was increased (*TLR4*: *p* = 0.0006) (*p-ERK*: *p* = 0.0327). These results indicated that Sr could exert the anti-inflammatory effects induced by an HFD via the *TLR4/p38 MAPK/ERK* and *NF-κB* pathways.

### 2.7. Sr Suppressed the Alterations in Many Aspects Related to Hippocampal Synaptic Plasticity Induced by an HFD

**Figure 7:**
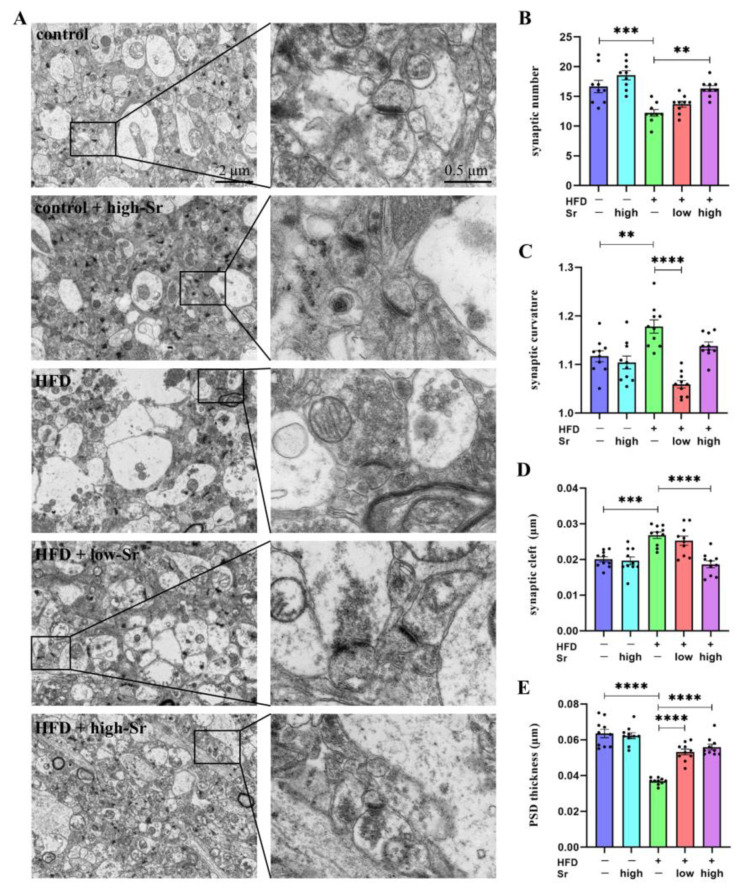
Sr suppressed the changes in synaptic plasticity in the hippocampus. (**A**) Representative images of synaptic ultra-structure. (**B**–**E**) Quantitative analysis of synaptic number (**B**), synaptic curvature (**C**), synaptic cleft (**D**), and PSD thickness (**E**) were detected through TEM ((**B**) *n* = 9 per group; (**C**–**E**) *n* = 10 per group). Data are presented as mean ± SEM. ** *p* < 0.01, *** *p* < 0.001, and **** *p* < 0.0001.

It was previously shown that the hippocampus is particularly vulnerable to damage due to neuroinflammation [39]. Because hippocampal synaptic plasticity is believed to be the neurobiological basis of learning memory and cognitive function, we examined alterations in the synaptic quantity and ultra-structure [40]. As shown in Figure 7A, TEM was performed to measure the ultra-structure of the synapses in the hippocampus, and the number of synapses was counted. The results found that the decreased synaptic number in the HFD-treated mice (*p* = 0.0005; Figure 7B) was restored by high Sr administration (*p* = 0.0015; Figure 7B). As displayed in Figure 7C,D, many aspects related to hippocampal synaptic plasticity, including the synaptic curvature and synaptic cleft, increased under the influence of an HFD (synaptic curvature: *p* = 0.0028) (synaptic cleft: *p* = 0.0002); the levels of these aspects were decreased by Sr treatment (low Sr: (synaptic curvature: *p* < 0.0001)) (high Sr: (synaptic cleft: *p* < 0.0001)). In addition, the postsynaptic density (PSD) thickness was reduced in the HFD group, and that was alleviated by Sr (HFD: *p* < 0.0001) (low Sr: *p* < 0.0001) (high Sr: *p* < 0.0001) (Figure 7E). Taken together, these findings indicated that Sr could suppress the alterations in hippocampal synaptic plasticity in the HFD mice.

## 3. Discussion

**Figure 8:**
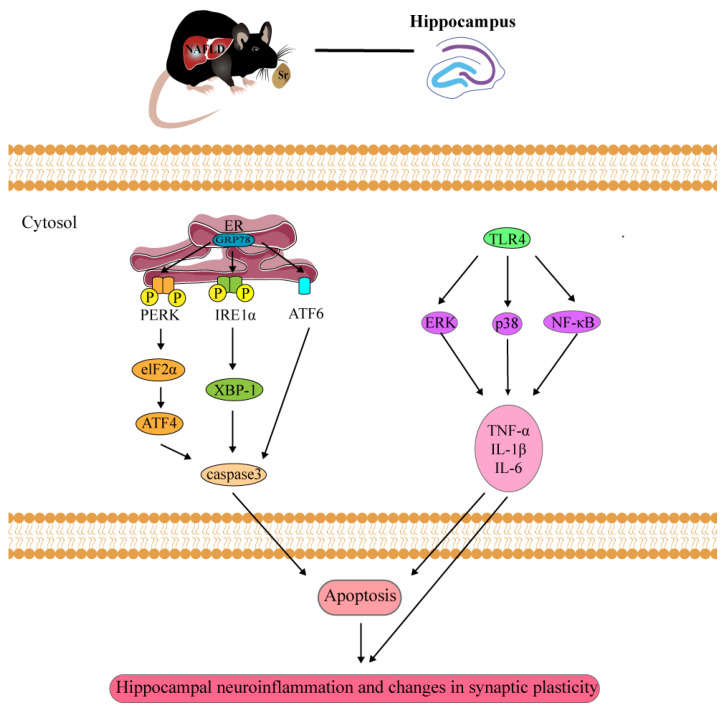
Schematic diagram of the protective effects and potential therapeutic mechanisms of Sr against HFD-induced hippocampal damage in the NAFLD mouse brain.

As far as we are aware, this is the first study to investigate the protective effects of Sr on NAFLD-induced hippocampal damage. The pathogenesis and development of NAFLD are quite complex processes involving multiple pathways, including imbalanced diets, lipotoxicity, oxidative stress, and inflammation [2]. It has been illustrated that NAFLD induced by an HFD not only induces inflammation and ERS [7,18,41] but also triggers cognitive deficits and decreased synaptic plasticity [42,43]. Accordingly, the evidence suggested that NAFLD might be a risk factor for CNS diseases [44]. Sr, a promising trace element in the body, triggers new bone formation through the induction of osteoblasts and the prevention of osteoclast activity [45]. In addition, Sr has been proven to be an anti-inflammatory drug with immunomodulatory properties [46,47]. The aim of this research was to investigate whether Sr could exert its neuroprotective effects by inhibiting the ERS, apoptosis, and neuroinflammation induced by an HFD in an NAFLD mouse model. We observed that Sr reduced the increased mRNA expression levels of *IL-1β*, *IL-6*, and *TNF-α* induced by an HFD. Similarly, the activation of microglia and astrocytes was inhibited by Sr administration in the hippocampus. In addition, the observed damage to the ultra-structural synaptic architecture induced by an HFD could be prevented by Sr administration in this study. Mechanism studies suggested that the inhibitory effects of Sr on HFD-induced hippocampal damage in the NAFLD mouse model were mediated by some of the signaling pathways relating to ERS or inflammation (Figure 8). This study implies that Sr has beneficial effects on repairing the damage to the hippocampus induced by an HFD in NAFLD mice.

To examine the function of Sr on the hippocampal damage induced by NAFLD, C57BL/6J mice were fed with an HFD to establish the NAFLD model in vivo. According to the previous study, the same or a similar dietary composition and duration were used to successfully induce liver steatosis, inflammation, and fibrosis [48,49,50]. In the present study, the mice in the model group showed an overall increase in body weight after 12 weeks of HFD feeding. The average weight gain trend appeared to be different in each group. However, it was not possible to regroup the mice because of the fights among them. It is worth noting that the significant weight gain and the large accumulation of fat in the liver and epididymis shown through a histological examination demonstrated the successful establishment of the NAFLD mouse model through HFD feeding.

C-Fos is an immediate-early gene that has been widely used as an indicator of neuronal activation [51]. In the current study, the c-Fos^+^ cells in the GCL decreased in the HFD mice. Similar data were previously reported that showed that an HFD prevented the enteric activation of c-Fos expression [52]. Because c-Fos is often expressed when neurons fire action potentials, c-Fos in the hippocampus can be used to assess the animal responses to stress [53]. Compared with the control group, the expression of c-fos was significantly decreased in the HFD group, which may be due to the inhibition of neuronal action potentials by a long-term HFD. Treatment with Sr promoted the expression of c-Fos in the HFD-fed mice, indicating that Sr alleviated the impacts of an HFD. However, the expression of c-Fos was also promoted by high Sr in normal mice. The observed increase in c-Fos could be attributed to Sr treatment. It is possible that Sr treatment promoted the neuronal activation of the hippocampus in the brain.

Previous studies have demonstrated that alterations in the expression of c-Fos are associated with ERS [54,55]. The occurrence and development of various long-term HFD-induced metabolic disorders, such as obesity, insulin resistance, and type II diabetes, are closely associated with sustained ERS [56]. Moreover, sustained ERS also has the potential to elicit inflammation and facilitate cell apoptosis [57]. In accordance with recent studies, our results indicated that an HFD could result in the accumulation of unfolded or misfolded proteins in the endoplasmic reticulum (ER) lumen, subsequently activating ERS and leading to apoptosis. When ERS occurs, the key ER regulatory protein *GRP78* dissociates from the ER transmembrane proteins, leading to the activation of the transmembrane proteins [58]. Compared with the control group, the expression of *GRP78* did not show a significant difference in the HFD group, but there was a certain upward trend. Apart from this, there were significant differences in some of the ERS-associated proteins that we measured. All three ER membrane sensors (*IRE1*, *PERK*, and *ATF6*), which activate the *IRE1*-spliced *XBP1*, *PERK-eIF2α-ATF4*, and *ATF6* pathways to the cleaved *ATF6* signaling pathway, respectively, induce downstream *CHOP*, a pro-apoptotic transcription factor [59,60,61]. The activation of *CHOP* induces *caspase-3*-mediated apoptotic signaling [62]. *Caspase-3* can also be activated in excessive ERS conditions [63]. As a key promoter of apoptosis, *caspase-3* activation is the final concentration point of all apoptotic pathways [24]. In this study, the *caspase-3* protein level was significantly increased in the hippocampus of the HFD mice, and, importantly, Sr treatment could decrease *caspase-3* protein expression. Therefore, we speculate that Sr-mediated anti-apoptosis effects contribute to the increased survival rate of cells in hippocampi under NAFLD.

Neuroinflammation is inextricably linked with apoptosis and ERS. Not unexpectedly, there was an increase in inflammatory cytokines in the HFD mice. In accordance with the present results, previous studies have demonstrated that neuroinflammation is one of the major symptoms of NAFLD and that it is involved in the progression of NAFLD [64]. Microglia and astrocytes are the resident immune cells of the CNS and are the primary mediators of neuroinflammation [65,66]. The proliferation of microglia and astrocytes is characterized as the classic inflammatory phenotype [67,68]. The increased densities of microglia and astrocytes in the HFD mice revealed that they became denser compared to those in the control group. Secreted factors from microglia are known to affect astrocytes and vice versa [69]. Surprisingly, we found that high Sr increased some inflammatory markers in the normal mice. The reason might be that Sr is a trace element, and the body does not normally ingest a high level of Sr. Therefore, high Sr might be a mild irritation in normal mice, leading to certain inflammatory responses, which was especially reflected in *TNF-α* expression. According to our findings, the release of *IL-1β*, *IL-6*, and *TNF-α* was decreased in the HFD mice with Sr treatment. Hence, it could conceivably be hypothesized that the hippocampal activation of microglia and astrocytes induced by an HFD was suppressed by Sr administration. As expected, treatment with Sr reversed the density changes induced by an HFD, and the density results of the Sr-treated group were almost close to those of the control group, suggesting that Sr played an anti-inflammatory role via modulating the activation of microglia and astrocytes. We further investigated the mechanism by which Sr may inhibit the HFD-induced hippocampal inflammation mediated by the *TLR4/p38 MAPK/ERK* and *NF-κB* signaling pathways. It has been demonstrated that the *HMGB1/TLR4/NF-κB* axis is the critical pro-neuroinflammatory signaling pathway in the CNS [70]. After the activation of *TLR4*, the recruitment and promotion of *NF-κB* occurs in neuroinflammation [71]. *TLR4* results in the production of inflammatory factors through phosphorylation of *p38*, *ERK*, *JNK* as well as the activation of *NF-κB* [72,73]. Our results revealed that an HFD increased the expression of phosphorylated *p38* and *NF-κB* in the hippocampus, and Sr inhibited the above increases. According to this, we demonstrated that Sr exerted its protective effects via suppressing the phosphorylation of *p38* and *NF-κB*, which subsequently led to reductions in *IL-1β*, *IL-6*, and *TNF-α* in the mice that were fed an HFD. This may explain why Sr suppressed the activation of microglia and astrocytes induced by an HFD in the NAFLD mice.

CNS neuroinflammatory damage has become a crucial reason for learning/memory impairment [74]. It has been widely recognized that an HFD leads to neuroinflammation in the hippocampus and decreased learning and memory functions [75]. The structural and functional plasticity of the synapses is critical for learning and memory [76]. The plasticity of the synaptic structure is mainly reflected in the ultra-structural changes in the synaptic number, synaptic curvature, synaptic cleft, and PSD thickness [77]. Our results showed that an HFD not only increased the synaptic curvature and synaptic cleft but also decreased the synaptic number and PSD thickness. It was possible that the increases in the synaptic curvature in the HFD group were due to compensatory effects. The most interesting finding was that Sr could inhibit these changes. Based on these findings, we indicated that Sr treatment could prevent HFD-induced damage to the ultra-structural synaptic architecture in the NAFLD model. Additionally, Sr had a protective effect on the hippocampal damage caused by NAFLD.

In summary, our results suggested that an HFD induced both ERS and inflammatory responses in the mice, involving a decrease in the c-Fos^+^ cell level, the activation of microglia and astrocytes, and increases in the pro-inflammatory cytokine levels. However, Sr did not only inhibit ERS through three *UPR* signaling pathways (the *IRE1α-XBP1*, *PERK-eIF2α-ATF4*, and *ATF6* pathways) but also inhibited the inflammation induced by an HFD via blocking the *TLR4/p38 MAPK/ERK* and *NF-κB* pathways. In addition, Sr could suppress alterations in hippocampal synaptic plasticity in the NAFLD mice. The protective effect of Sr on hippocampal damage may be mediated by alleviating ERS and the anti-inflammatory effects, which has the potential to become a new therapeutic approach to prevent and treat NAFLD-induced hippocampal damage.

## 4. Materials and Methods

### 4.1. Mice, Diets, and Treatments

Male C57BL/6J mice aged six weeks were purchased from Chongqing Byrness Weil biotech Ltd. (Chongqing, China). The mice were housed in a standard rodent room (temperature of 22–26 °C, relative humidity of 40–70%, and 12 h day/night cycle), and body weight was assessed weekly. Before the formal experiment, adaptive feeding was implemented with standard mouse chow and water intake for a week. The mice were randomly divided into 5 groups (*n* = 12 per group) and housed in cages (*n* = 4 per cage) after one week of adaptive feeding as follows (Figure 1A):

(1) The control group was fed standard mouse chow for 18 weeks.

(2) The control + high-Sr group was fed standard mouse chow for 12 weeks. Then, the mice were fed standard mouse chow mixed with SrCl_2_·6H_2_O (750 mg/kg of Sr) for 6 weeks.

(3) The HFD group was fed a 45% high-fat diet for 18 weeks.

(4) The HFD + low-Sr group was fed a 45% high-fat diet for 12 weeks. Then, the mice were fed a 45% high-fat diet mixed with SrCl_2_·6H_2_O (250 mg/kg of Sr) for 6 weeks.

(5) The HFD + high-Sr group was fed a 45% high-fat diet for 12 weeks. Then, the mice were fed a 45% high-fat diet mixed with SrCl_2_·6H_2_O (750 mg/kg of Sr) for 6 weeks.

The standard mouse chow (AIN005, 20% protein, 70% carbohydrate, and 10% fat) and the 45% high-fat diet (HD004, 20% protein, 35% carbohydrate, and 45% fat) were purchased from Beijing Botai Hongda Biotechnology Co. The SrCl_2_·6H_2_O (V900279) was purchased from Shanghai Sigma–Aldrich. The mouse chow was abraded to 1~5 mm particles using sample mill (SMF2002, Supor, Hangzhou, China). The SrCl_2_·6H_2_O was dissolved in distilled water and mixed evenly with diets (dissolve in 10 mL distilled water in per kg control diets and 5 mL in per kg HFD diets). Then, the Sr-feed mixture was pressed into cylindrical chow using feed pellet mill. The chow was then stored at −20 °C separately after drying. Animal investigators performing the experiments were blinded to the group assignment of mice during the experiment. Animal experiments were conducted according to the Guide for Care and Use of Laboratory Animals: Northwest A&F University, and they were approved and performed in accordance with the Animal Care Commission of the College of Veterinary Medicine, Northwest A&F University (Approval No. 2021051).

### 4.2. Histology

Mouse livers and epididymal adipose were dissected from the control mice and the HFD mice. Livers were placed in 30% sucrose solution in PB at 4 °C overnight before being placed in optimal cutting temperature compound and being frozen in liquid nitrogen. Liver samples underwent frozen sectioning at 8 μm thickness and Oil Red O staining to assess hepatic fat accumulation. Hematoxylin counterstaining was performed to visualize nuclei. Epididymal adipose was fixed in 10% neutral formalin, processed in paraffin blocks, sectioned to a thickness of 20 μm, and stained with hematoxylin and eosin. These frozen sections and paraffin sections were observed and photographed under a microscope (Nikon, Tokyo, Japan). The Oil Red-O staining area and adipocyte size were quantified using ImageJ software (Fiji, https://imagej.net/software/fiji/, accessed on 28 May 2023).

### 4.3. Western Blot

The brains of mice were rapidly dissected on ice after being sacrificed, and the hippocampus tissues were isolated. The hippocampal samples were homogenized in RIPA lysis buffer containing a protease inhibitor cocktail (P1400) and phosphatase inhibitor cocktail. Protein concentrations were determined with the BCA Protein Assay Kit (Solarbio, Beijing, China). Briefly, the protein samples (20 μg each) were separated through sodium dodecyl sulfate–polyacrylamide gel electrophoresis and were transferred onto polyvinylidene difluoride (PVDF) membranes. We blocked the PVDF membranes for 2 h at room temperature using 5% skim milk (skim milk dissolved in TBST (TBS containing 0.1% Tween-20)). Subsequently, the following primary antibodies were used to incubate the membranes overnight at 4 °C: rabbit anti-*NF-κB* (#8242), rabbit anti-*p38* (#9212), rabbit anti-*ERK* (#9102), rabbit anti-phospho-*ERK* (*p-ERK*, #4370), rabbit anti-phospho-*p38* (*p-p38*, #4511), and anti-*caspase-3* (#9662) (purchased from Cell Signaling Technology (Danvers, MA, USA)); mouse anti-*ATF6* (EM1701-94) (purchased from Hangzhou Huaan Biotechnology Co., Ltd., Hangzhou, China); rabbit anti-*XBP1* (A1731), rabbit anti-phospho-*NF-κB* (*p- NF-κB*, AP0475), rabbit anti-*GRP78* (A0241), and mouse anti-*β-actin* (purchased from Wuhan ABclonal Technology Co., Ltd., Wuhan, China); rabbit anti-*eIF2α* (ab115822), rabbit anti-*TLR4* (ab13556), and rabbit anti-phospho-*eIF2α* (*p-eIF2α*, ab32157) (purchased from Abcam (Cambridge, MA, USA)); and rabbit anti-*CHOP* (BM4962), anti-phospho-*IRE1α* (*p-IRE1α*, BM4444), rabbit anti-*ATF4* (BM5179), and rabbit anti-*IRE1α* (A00683-1) (purchased from Wuhan BOSTER Biological Technology Co., Ltd., Wuhan, China). The membranes were incubated with horseradish peroxidase (HRP)-conjugated goat anti-rabbit IgG antibody or goat anti-mouse IgG antibody (Cell Signaling Technology) at 4 °C for 5 h and were washed with TBST. The blots were developed with ChemiDoc™ MP Imaging System (Bio-Rad, Hercules, CA, USA) using an enhanced chemiluminescence detection kit (GE Healthcare, Buckinghamshire, UK), and relative band densities were quantified using ImageJ software (Fiji, https://imagej.net/software/fiji/, accessed on 28 May 2023).

### 4.4. Quantitative RT-PCR

Quantitative RT-PCR analysis was essentially performed as previously described [78]. Total RNA was isolated from hippocampus using TRIzol reagent (Invitrogen, Waltham, MA, USA). cDNA was synthesized from 2 μg of total RNA using the FastKing RT Kit (TIANGEN Biotech, Beijing, China) according to manufacturer’s instructions. The synthesized cDNA was used as the RT-PCR template. This was performed on a Bio-Rad CFX96TM real-time PCR detection system (Bio-Rad, Hercules, CA, USA) using NovoStart^®^ SYBR qPCR SuperMix Plus (Novoprotein Scientific Inc., Nanjing, China). The primers used were as follows. *GAPDH*: forward primer was 5′-AGGTTGTCTCCTGCGACTGCA-3′, and reverse primer was 5′-GTGGTCCAGGGTTTCTTACTCC-3′. *IL-6*: forward primer was 5′-CACTTCACAAGTCGGAGGCT-3′, and reverse primer was 5′-CTGCAAGTGCATCATCGTTGT-3′. *IL-1β*: forward primer was 5′-TGACGGACCCCAAAAGATGA-3′, and reverse primer was 5′-TCTCCACAGCCACAATGAGT-3′. *TNF-α*: forward primer was 5′-AGTCCGGGCAGGTCTACTTT-3′, and reverse primer was 5′-GTCACTGTCCCAGCATCTTGT-3′. *GAPDH* was used as an internal control gene. The relative quantification of mRNA expression was calculated with the 2^−ΔΔCT^ method [79].

### 4.5. Immunofluorescence Staining

We anesthetized the mice with 0.56% sodium pentobarbital and transcardially perfused the mice with 0.9% saline followed by 4% paraformaldehyde (PFA, pH 7.4). All mice brains were obtained and post-fixed in 4% PFA in 0.1 M phosphate buffer (PB, pH 7.4) at 4 °C for over 72 h. The fixed brains were sectioned using a microtome (VT1000S, Leica, Wetzlar, Germany) into coronal slices (thickness of 50 µm). After rinsing three times in PB, sections were incubated with primary antibodies in blocking solution (4% BSA, 1% NGS, and 0.3% Triton in phosphate buffer) for 24 h at 4 °C. The following primary antibodies were used: rabbit anti-c-Fos, rabbit anti-ionized calcium-binding adapter molecule 1 (Iba1), and rabbit anti-glial fibrillary acidic protein (GFAP). The sections were washed three times before incubation with secondary antibodies overnight at 4 °C in the darkness. The secondary antibody used in this study was Alexa Fluor 647 goat anti-rabbit IgG (ab150079, 1:500, Abcam). DAPI was used to stain the nuclei. The slices were washed three times with PB and mounted with Fluorescence Mounting Medium (Dako, Carpentaria, Australia). Stained slides were photographed using a structured illumination microscope (Zeiss Axio Observer Z1, Zeiss, Oberkochen, Germany). The immunofluorescent images were analyzed and processed using ImageJ software.

### 4.6. Transmission Electron Microscopy (TEM)

Samples of hippocampus tissues (1 mm × 1 mm × 1 mm) were carefully collected to minimize mechanical damage, then primarily fixed in Fixative for TEM (G1102, Servicebio, Wuhan, China) at 4 °C. The sample avoided light after being fixed with 1% OsO_4_ (Ted Pella Inc., Redding, CA, USA) in 0.1 M PB (pH 7.4) for 2 h at room temperature. After removing OsO_4_, the tissues were rinsed in 0.1 M PB (pH 7.4) three times. After dehydrating at room temperature, resin penetration and embedding were performed; the embedding models containing the resin and samples were put into an oven at 65 °C for polymerization for over 48 h. After this, the resin blocks were removed from the embedded models and were placed at room temperature for standby application. Then, the resin blocks were cut into thin sections of 60–80 nm using an ultra-microtome (Leica UC7, Leica), and the sections were placed on 150-mesh cuprum grids using formvar film. The sections were stained with 2% uranium acetate saturated alcohol solution and 2.6% lead citrate and observed through TEM (HT7800, Hitachi, Tokyo, Japan).

### 4.7. Statistical Analysis

GraphPad Prism 8.0 software (San Diego, CA, USA) was used to analyze data, which are expressed as the mean ± standard error of the mean (SEM). The comparisons between groups were performed through one-way analysis of variance (ANOVA) and Tukey’s multiple comparison test. Significant differences were determined when *p* value was less than 0.05. Analyses were performed by an investigator blinded to the experimental conditions.

## Data Availability

The data presented in this study are available on request from the corresponding author.
